# A visual processing advantage for young-adolescent deaf observers: Evidence from face and object matching tasks

**DOI:** 10.1038/srep41133

**Published:** 2017-01-24

**Authors:** Ahmed M. Megreya, Markus Bindemann

**Affiliations:** 1 Department of Psychological Sciences, College of Education, Qatar University, Qatar; 2 School of Psychology, University of Kent, UK

## Abstract

It is unresolved whether the permanent auditory deprivation that deaf people experience leads to the enhanced visual processing of faces. The current study explored this question with a matching task in which observers searched for a target face among a concurrent lineup of ten faces. This was compared with a control task in which the same stimuli were presented upside down, to disrupt typical face processing, and an object matching task. A sample of young-adolescent deaf observers performed with higher accuracy than hearing controls across all of these tasks. These results clarify previous findings and provide evidence for a general visual processing advantage in deaf observers rather than a face-specific effect.

It has been known for many years that the permanent loss of sight can improve a wide range of fundamental auditory processes, such as sound localization^1^, pitch discrimination^2^, voice perception^3^, and auditory memory^4^. In contrast, it remains unresolved whether deaf individuals experience enhanced visual perception in comparison to hearing persons (for reviews, see refs 5 and 6). According to the *perceptual deficit hypothesis*, an impaired sense might impair the development of other senses^7^, which suggests that deaf individuals might also present visual processing deficits. In contrast, the *sensory compensation hypothesis* posits that the impairment of one sense can cause compensatory proficiencies in other senses^8^, which might suggest a visual processing superiority in deaf individuals. Studies of visual processing in deaf participants have yielded inconsistent results, demonstrating either an advantage for deaf individuals^9^,^10^,^11^,^12^, or hearing persons^11^,^13^,^14^,^15^, or equivalent performance for both groups of observers^10^,^11^,^16^,^17^. These inconsistencies do not appear to arise from differences between the participant samples of these studies or visual stimulus characteristics, but have been linked to target eccentricity and selective visual attention^5^,^6^, whereby processing might be enhanced in deaf observers particularly for tasks requiring peripheral visual attention. It has also been suggested that deaf individuals perform better on tasks that require a global perceptual strategy, in which stimuli are processed as coherent gestalts, whereas hearing individuals are advantaged in analytical perceptual strategies that focus on specific details or features^18^.

The present study examined the effect of hearing impairment on a visual task that is held to depend strongly on global processing - the perception of faces (see, e.g., refs 19, 20, 21, 22). Very few studies have examined face processing in deaf individuals, and have produced mixed results. In the Benton Face Recognition Test (BFRT: ref. 23), which requires observers to perceptually match a target to a six-person lineup, deaf participants exhibited a performance advantage over hearing participants^24^. However, this advantage was not found when good-quality frontal or profile faces were shown, for which performance was at or close to ceiling, but only when task difficulty was increased substantially by obscuring facial features through the addition of shadows. This specific advantage for shadow faces was abolished by presenting these faces upside-down, which is held to disrupt global face processing (see, e.g., refs 20, 21, 22). This could indicate that the partial face advantage of deaf observers might arise from specific face processing mechanisms rather than a general improvement in visual discrimination. However, an alternative explanation also exists, as performance for inverted shadow faces was close to chance. This raises the possibility that this condition was not sufficiently sensitive to reveal differences between groups.

Other studies produced similarly mixed results. In a recognition memory task, in which observers had to identify previously seen faces, deaf and hearing participants displayed comparable accuracy^25^. Performance for both groups was also similar during the classification of sex and age from impoverished black-and-white illustrations of (Mooney) faces, which have been used to assess the configural processing of faces, and for the detection of changes to the eyes and nose regions of face portraits^25^. However, a detection advantage was found in deaf observers for changes to the mouth region.

A face advantage has also been observed in another memory task, in which observers had to search a spread-out deck of upside-down cards for pairs of identical faces and objects. In this task, deaf participants displayed a search advantage over hearing participants for images of faces that was not present for verbalizable non-face objects (e.g., a bed or cup; see ref. 26). However, this difference might reflect task difficulty, which was mismatched across conditions. In line with this reasoning, a comparable advantage to faces was found for objects that were taken from a single visual category (shoes) to equate differences in distinctiveness between stimuli^27^.

Taken together, these findings leave considerable doubt about the nature of a face processing advantage in deaf participants. All of these studies reported a face advantage in deaf observers^24^,^25^,^26^,^27^, but this was not always present across different face conditions^24^,^25^. Moreover, considering this advantage was not present for inverted faces^24^, but was found for highly similar non-face objects^24^,^27^,^28^,^29^, it is not clear whether it reflects a face-specific effect or a more generalized visual cognition advantage for stimuli that display high within-category similarity. These inconsistent results across studies might reflect the different tasks, but could also relate to small sample sizes, low trial numbers per condition, and close-to-ceiling performance in some experiments (see, e.g., ref. 24).

In light of these mixed findings, the current study sought to reexamine the face advantage in deaf participants. In contrast to previous studies, we employed a task that measures face encoding directly, by minimizing the contribution of memory demands (for reviews see refs 30 and 31), and that provides considerably more trials and produces average performance that is substantially below ceiling. In this task, observers were required to match an image of an unfamiliar target face to a ten-face line-up, in which a different image of the target could be present or absent^32^. This task was administered without time pressure, and all face images were high-quality same-day photographs that were presented in the same viewing angle, and under similar lighting and pose (e.g., for different examples, see refs 32, 33, 34). This task typically yields mean error rates of ~30% for target-present and target-absent line-ups (see refs 32, 33, 34), and produces a broad range in individual performance (see refs 35, 36, 37). This suggests that this is an appropriate task to assess the face recognition ability of deaf and hearing participants.

To determine whether any advantage in this task is specific to faces or part of a more generalized visual cognitive advantage, the present study followed *differential deficit* conceptualization^38^,^39^. Accordingly, a *differential superiority* can be conceptualized as a greater performance on one task than on other psychometrically-matched control tasks. The control tasks that were employed here comprised an inverted version of the face task, in which all stimuli were turned upside-down, to examine performance when typical face processes are disrupted^40^,^41^,^42^, and a 1-in-6 object matching task. Previous studies demonstrate strong positive correlations between the upright and inverted face tasks and between the face and object task^37^. These tasks are therefore appropriate for exploring the existence of face identification superiority in deaf individuals.

## Method

### Participants

Eighty Qatari students volunteered to participate in this experiment. These comprised 40 deaf students (24 females, 16 males; mean age = 13.3 years, SD = 3.2, with an age range of 8–22 years), who were proficient in Arab Sign Language, and 40 hearing students (25 females, 15 males; 13.5 years, SD = 3.2, with an age range of 8–20 years), who were all non-signers. Of the deaf sample, 19 students were congenitally deaf (47.5%), nine students suffered early hearing loss before the age five (22.5%), and 12 students had profound hard hearing that required a hearing aid apparatus (30%). The two groups of participants were matched in gender, χ^2^ (1) < 1, *p* > 0.05, and age, *t* (78) < 1, *p* > 0.05. All participants reported normal or corrected to normal vision. Written informed consent for participation was obtained from the participants’ parents. Ethical approval for the experiment was provided by Qatar University’s institutional review board (QU-IRB) and all methods were performed in accordance with the QU-IRB guidelines and regulations.

### Stimuli

The face-matching task comprised stimulus arrays for sixty target identities. Each array consisted of a video still of a face target and an identity lineup comprising digital photographs of ten faces, which were shown underneath the target. For each target face, two accompanying lineups were created, in which the target identity was either present or absent, yielding a total of 120 stimulus arrays. Notably, target and array photos were taken with two different high-quality cameras to eliminate image matching, but on the same day and under similar lighting conditions to eliminate superficial differences in appearance, such as hairstyle. All face images measured approximately 7 × 10 cm and were shown in full-face view with a neutral expression (for full details about the construction of these stimuli, see ref. 30). A complementary set of 120 inverted face arrays were created for the control condition by turning the stimuli upside-down (for example stimuli of the upright arrays, see refs 30 and 40).

The stimuli for the object-matching task were adapted from the Matching Familiar Figures Test (MFFT). This test consists of 20 line drawings of common objects, which act as targets and are presented above six variants of the same object, only one of which is exactly identical to the target image (for example stimuli, see ref. 40).

### Procedure

Participants were tested individually with a MacPro laptop and SuperLap software. In the face-matching task, each participant completed 60 trials, comprising 30 target-present arrays (15 upright, 15 inverted) and 30 target-absent arrays (15 upright, 15 inverted), which were presented in a random order. Four versions of this task were constructed to counter-balance presence (present/absent) and orientation (upright/inverted) of each target across participants. For each array, participants were asked to decide whether the target was present in the lineup below, and if so, to indicate which of the faces they believed it to be by pressing numbered keys on the computer keyboard. Each stimulus array was presented onscreen until a response was made.

In the object-matching task, participants were asked to find the object in the six-item array that matched the target. Each participant completed 20 randomized trials, which were presented until a response was made on the computer keyboard. The order of the face and object tasks was counterbalanced across participants. Both tasks were self-paced and participants were encouraged to perform as accurately as possible.

## Results

### Accuracy

Consistent with previous studies^33^,^34^,^37^,^43^, five measures were calculated to assess performance on the face matching task. For target-present arrays, we calculated correct identifications (selection of the correct face from a lineup), misses (the decision that a target is absent from a lineup despite its presence), and misidentifications (selection of a wrong lineup face as a target). For target-absent trials, we calculated correct rejections (the correct decision that the target is not present). In addition, overall accuracy was calculated by taking the mean of correct identifications and correct rejections, to illustrate accuracy when this is combined for target-present and target-absent trials. A series of one-way between-subject ANOVAs revealed no differences between participants with congenital deafness, early-year deafness, and profound hard hearing using all of these five face matching measures, all *Fs*(2,39) ≤ 1. Consequently, the data from these participants were collapsed for all subsequent analyses.

**Figure 1:**
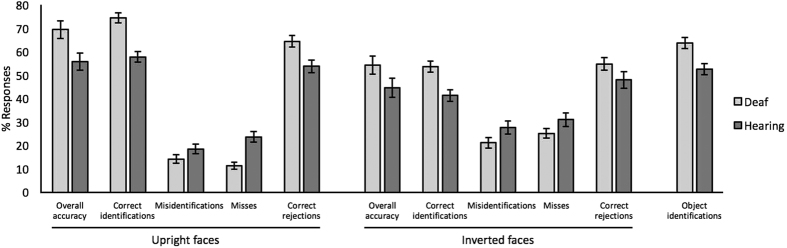
Performance for deaf and hearing observers on the face and object matching tasks. Error bars show standard error of the means.

Figure 1 shows the cross-subject mean percentages for the five face-matching measures. These were subjected to separate 2 (hearing *vs.* deaf participants) × 2 (upright *vs.* inverted face orientation) mixed-factor ANOVAs. For overall accuracy, ANOVA revealed an interaction between hearing and face orientation, *F*(1,78) = 5.15, *p* < 0.05, 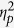 = 0.06. Analysis of simple main effects showed that deaf and hearing participants matched upright faces more accurately than inverted faces, *F*(1,78) = 143.56, *p* < 0.001, 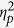 = 0.65 and *F*(1,78) = 76.97, *p* < 0.001, 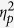 = 0.50, respectively. However, deaf participants outperformed hearing participants in the upright, *F*(1,156) = 25.02, *p* < 0.001, 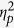 = 0.14, and inverted conditions, *F*(1,156) = 12.30, *p* < 0.001, 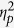 = 0.07.

For correct identifications and correct rejections, main effects of hearing were found, *F*(1,78) = 25.55, *p* < 0.001, 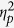 = 0.55, and *F*(1,78) = 5.30, *p* < 0.05, 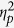 = 0.43, due to higher accuracy for deaf participants. A main effect of face orientation was also present for both measures, *F*(1,78) = 157.63, *p* < 0.001, 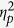 = 0.67, and *F*(1,78) = 46.06, *p* < 0.001, 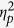 = 0.37, as accuracy was best for upright face stimuli. The interactions were not significant, *F*(1,78) = 2.12, *p* = 0.15, 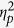 = 0.03, and *F*(1,78) = 2.83, *p* = 0.10, 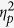 = 0.03, respectively.

For the error measures for face-present trials, a main effect of hearing was not found for misidentifications, *F*(1,78) = 3.34, *p* = 0.07, 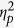 = 0.18, but was present for misses, *F*(1,78) = 9.85, *p* < 0.01, 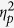 = 0.35, due to lower miss rates in deaf participants. A main effect of face orientation was also present for both measures, *F*(1,78) = 57.74, *p* < 0.001, 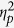 = 0.42, and *F*(1,78) = 38.99, *p* < 0.001, 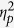 = 0.33, as misses and misidentifications were lowest for upright faces. In addition, there was an interaction of hearing and face orientation for misses, *F*(1,78) = 5.44, *p* < 0.05, 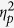 = 0.06, but not for misidentifications, *F*(1,78) = 0.70, *p* = 0.40, 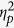 = 0.01. Analysis of simple main effects showed that deaf and hearing participants recorded more misses when faces were presented upside-down, *F*(1,78) = 49.32, *p* < 0.001, 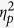 = 0.39, and *F*(1,78) = 13.86, *p* < 0.001, 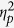 = 0.15, respectively. In addition, deaf participants recorded fewer misses than hearing observers in the inverted face condition, *F*(1,156) = 14.74, *p* < 0.001, 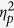 = 0.09, but not in the upright face condition, *F*(1,156) = 3.30, *p* = 0.07, 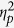 = 0.02.

Figure 1 also illustrates performance in the object-matching task. As for upright and inverted faces, deaf participants were more accurate in this task than hearing participants, *t*(78) = 3.38, *p* < 0.001, Cohen’s *d* = 0.74.

### d prime and criterion

For the face identification task, correct identifications and false positives (i.e., mistaken identifications on target-absent trials, and which are the inverse of correct rejections) were also converted into the signal detection measures of *d*′ and *criterion*. Similar to accuracy, *d*′ revealed an interaction between hearing and face orientation, *F*(1,78) = 6.40, *p* < 0.05, 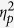 = 0.07. Simple main effects showed that deaf participants outperformed controls in the upright face condition, *F*(1,78) = 26.05, *p* < 0.001, 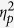 = 0.14 (mean *d*′ for deaf = 1.14 vs. controls = 0.33), and the inverted face condition, *F*(1,78) = 11.69, *p* < 0.001, 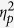 = 0.07 (mean *d*′ for deaf = 0.25 vs. controls = −0.28). In addition, *d*′ was enhanced for upright over inverted faces in the deaf sample, *F*(1,78) = 141.58, *p* < 0.001, 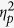 = 0.64, and the control group, *F*(1,78) = 69.22, *p* < 0.001, 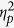 = 0.47. For *criterion,* a main effect of face orientation was found, *F*(1,78) = 34.75, *p* < 0.001, 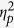 = 0.31, as participants were more likely to commit to an identification in the upright compared to the inverted face condition. No main effect of hearing, *F*(1,78) = 1.93, *p* = 0.17, 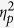 = 0.10, and no interaction between face orientation and hearing were found, *F*(1,78) < 1, *p* = 0.54, 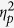 = 0.01.

### Response times

**Figure 2:**
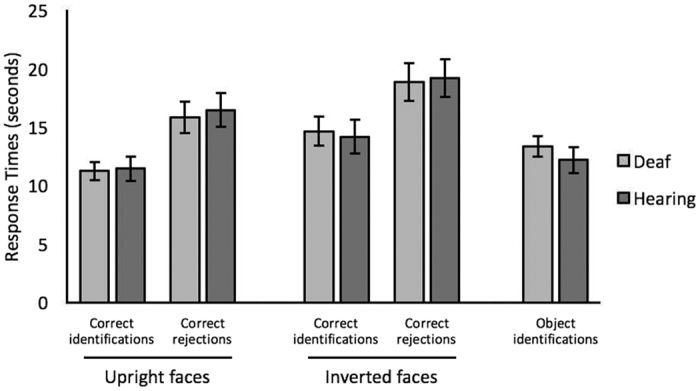
Response times for correct responses of deaf and hearing observers on the face and object matching tasks. Error bars show standard error of the means.

Although accuracy was emphasized, response times for correct responses were analyzed for completeness (see Fig. 2). In the face matching task, these data were subjected to two 2 × 2 mixed-factor ANOVAs for correct identifications and correct rejections, which showed no main effects of hearing, *F*s < 1, 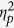 ≤ 0.02. A main effect of face orientation was found for correct identifications, *F*(1,78) = 51.07, *p* < 0.001, 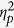 = 0.40, and correct rejections, *F*(1,78) = 47.15, *p* < 0.001, 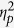 = 0.37. There was no interaction between hearing and face orientation using both correct identifications and correct rejections, both *F*s < 1, 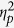 ≤ 0.04.

In the object-matching task, response times were comparable for deaf and hearing participants, *t*(78) < 1, *p* = 0.40, Cohen’s *d* = 0.19.

### Correlations between tasks

Pearson’s coefficient correlations were conducted to explore associations between the face and object-matching task in deaf and hearing participants. These data showed that all accuracy and RT measures for the face and objects tasks correlated in deaf participants, all *rs* ≥ 0.46, all *ps* < 0.001, and controls, all *rs* ≥ 0.31, all *ps* < 0.001.

## Discussion

This study examined whether a sample of young-adolescent deaf observers exhibit an advantage in face matching over hearing controls. Such an advantage was found in overall accuracy as well as in correct identifications and correct rejections of lineups, indicating a consistent effect. However, a similar advantage was also obtained for inverted faces, which provide identical visual content but for which typical face processes are held to be disrupted^20^,^21^,^22^, and in the object matching task.

These findings help to clarify the results of previous studies. Whereas these studies emphasize the existence of a face advantage in deaf observers, such an advantage was, in fact, more likely to be absent than present across different face tasks^25^ and different face conditions^24^,^25^. Moreover, the absence of a face advantage in previous studies could reflect ceiling performance^24^, whereas its presence could be attributed to differences in task demands between face and non-face objects^26^. However, a general visual processing advantage, for both face and non-face objects, was found when task demands were comparable for these different types of stimuli^27^. The current results converge with these findings by demonstrating an advantage for deaf observers in the processing of upright faces and their inverted counterparts, as well as for non-face objects in a matching task. All of these tasks were psychometrically-matched, and also correlated strongly, to provide clear evidence against *differential superiority*^38^,^39^ for face processing in deaf observers. Instead, these findings indicate a general visual processing advantage in deaf over hearing participants.

A simple explanation for these findings could be that deaf participants were more motivated and invested more effort in this task. The response times, which were matched for deaf and hearing participants, indicate this as unlikely. By comparison, other participant samples, such as passport officers, appear to invest particular effort in face identification, as indexed by longer response times, but still do not perform better than control participants^44^. We therefore suggest that motivation and effort are unlikely to account for the current results. However, these results might have implications for professions that rely on face identification, such as passport control^44^, or comparable tasks with non-face stimuli^45^,^46^, by indicating that deaf participants might be particularly suited for these by virtue of their enhanced visual skills. Future studies need to examine this suggestion.

We note that the deaf participants in the current study were all experienced in sign language, whereas only non-signing hearing controls were tested. The contrast of these groups produced the clearest differences in previous attempts to investigate enhanced face processing in deaf participants. Consequently, however, the question arises of whether a visual processing advantage for deaf observers reflects auditory deprivation or the experience of sign language. Some of the previous research reports an advantage for deaf over both signing and non-signing hearing observers, which performed at the same level^25^. However, this advantage was only present in one of three reported experiments (the facial feature recognition task), and only in one of three conditions in this task (the mouth detection condition). In other studies, hearing signers performed either more similarly to^26^,^27^ or identical to deaf signers than hearing non-signers^24^. Thus, it remains unclear whether the visual processing advantage might reflect additive effects of deafness and the long use of sign language, or a visual advantage that is driven primarily by the latter.

## Additional Information

**How to cite this article**: Megreya, A. M. and Bindemann, M. A visual processing advantage for young-adolescent deaf observers: Evidence from face and object matching tasks. *Sci. Rep.* **7**, 41133; doi: 10.1038/srep41133 (2017).

**Publisher's note:** Springer Nature remains neutral with regard to jurisdictional claims in published maps and institutional affiliations.
