# Erratum: A visual processing advantage for young-adolescent deaf observers: Evidence from face and object matching tasks

**DOI:** 10.1038/srep43497

**Published:** 2017-02-24

**Authors:** Ahmed M. Megreya, Markus Bindemann

Scientific Reports 7: Article number: 4113310.1038/srep41133; published online: 01 24 2017; updated: 02 24 2017

This article contains typographical errors in the ‘Methods’ section under subheading ‘Stimuli’.

“All face images measured approximately 7 × 10 cm and were shown in full-face view with a neutral expression (for full details about the construction of these stimuli, see ref. 30). A complementary set of 120 inverted face arrays were created for the control condition by turning the stimuli upside-down (for example stimuli of the upright arrays, see refs 30 and 40).

The stimuli for the object-matching task were adapted from the Matching Familiar Figures Test (MFFT). This test consists of 20 line drawings of common objects, which act as targets and are presented above six variants of the same object, only one of which is exactly identical to the target image (for example stimuli, see ref. 40)”.

should read:

“All face images measured approximately 7 × 10 cm and were shown in full-face view with a neutral expression (for full details about the construction of these stimuli, see ref. 33). A complementary set of 120 inverted face arrays were created for the control condition by turning the stimuli upside-down (for example stimuli of the upright arrays, see refs 33 and 38).

The stimuli for the object-matching task were adapted from the Matching Familiar Figures Test (MFFT). This test consists of 20 line drawings of common objects, which act as targets and are presented above six variants of the same object, only one of which is exactly identical to the target image (for example stimuli, see ref. 38)”.

